# The Influencing Factors of Breastfeeding for Full-Term Singleton Within Six Months of Birth in Xi'an Before the Covid-19 Pandemic

**DOI:** 10.3389/fped.2021.801436

**Published:** 2022-03-10

**Authors:** Zhan-Wang Yuan, Li Ma, Wen-Li Ge, Xiao-Ying Li, Xiao-Qin Zhang, Jing-Jie Zeng, Jun Yang, Peng-Fei Qu

**Affiliations:** ^1^Department of Nursing Care (Nursing Department), Northwest Women's and Children's Hospital, Xi'an, China; ^2^Department of President's Office, Northwest Women's and Children's Hospital, Xi'an, China; ^3^Department of Maternity (Maternity Department), Northwest Women's and Children's Hospital, Xi'an, China; ^4^Outpatient Department, Xi'an Gaoxin Hospital, Xi'an, China; ^5^Medical Department, Xi'an Huyi District Women's and Children's Health Family Planning Service Center, Xi'an, China; ^6^Translational Medicine Center, Northwest Women's and Children's Hospital, Xi'an, China

**Keywords:** Xi'an, exclusive breastfeeding, influencing factors, body weight gain, full-term infants

## Abstract

**Objective:**

The study is designed to understand the situation of full-term infants breastfeeding within 6 months of birth in Xi'an before the Covid-19 pandemic and analyze the influencing factors of exclusive breastfeeding.

**Methods:**

Five hospitals in Xi'an province have been selected as research centers. Full-term infants who met the inclusion and exclusion criteria were recruited from these centers between January 1 and February 28, 2019. The feeding situation at 10 days, 42 days, 3 months, and 6 months after birth were investigated. A self-designed breastfeeding questionnaire was used for investigation and follow-up. SPSS 22.0 was applied for statistical analysis of the data.

**Results:**

The exclusive breastfeeding rate of full-term infants on days 10 and 42 and at months three and six after birth was 61.38%, 54.78%, 48.83%, and 38.78%, respectively, with a decreasing trend over time. During breastfeeding within 48 h after delivery, 1,653 cases (91.83%) of puerpera had different grades of pain, including 1,325 cases (80.16%) of mild discomfort, 321 cases (19.42%) of moderate pain, and seven cases (0.42%) of severe pain. Within 24–48 h postpartum, 1,607 (89.27%) mothers faced problems related to postpartum breastfeeding. Among them, 694 (43,19%) neonates could not be fed effectively; 665 (41.38%) mothers had wound pain and had inconvenience to turn over; 598 (37.21%) neonates were difficult to wake up; 439 (27.32%) mothers had incorrect feeding posture; 181 (11.26%) mothers experienced other problems. The Cox risk regression model showed that weight gain during pregnancy was higher than the recommended standard. Living in suburban counties was a risk factor of exclusive breastfeeding for full-term infants. Participation in breastfeeding courses during pregnancy, feeding more than eight times daily after delivery, were the protective factors of exclusive breastfeeding for full-term infants.

**Conclusion:**

The body weight gain of parturients should be controlled within a reasonable range during pregnancy. Parturients were encouraged by medical staff to participate in breastfeeding courses or watch the breastfeeding process during pregnancy to increase their self-confidence and improve the rate of exclusive breastfeeding for full-term infants. In addition, it is necessary to strengthen the publicity of breastfeeding in suburban areas to promote breastfeeding.

## Background

The death of about two-thirds of children <5 years of age are caused by infections, and about 35% are related to malnutrition worldwide (1). In contrast, the death of only 21.4% of infants in 2008 in China was infection-related, of which pneumonia was 16.5%, diarrhea 3%, and neonatal septicemia 1.9% (2). However, child mortality is much higher in poor western provinces, and the incidence of mortality in children <5 years of age in the poorest areas is more than six times that in large cities (3). Breastfeeding has been demonstrated to reduce the occurrence and mortality of infants due to infection and reduce the risk of being overweight and obese (4). The World Health Organization recommended that exclusive breastfeeding (EBF) last 6 months and beginning complementary feeding at 6 months with continued breastfeeding up to 2 years and beyond (5). The “Children's Development Program 2011–2020 in China” sets a goal that the rate of EBF of infants from 0 to 6 months should be over 50% (6). Approximately 48% of infants start breastfeeding within the first hour after birth worldwide, excluding China. The statistical data from 2014 to 2020 showed that 44% of infants between 0 and 5 months old had EBF, including China (7).

The rate of EBF in China within 6 months varies among different regions. Although it shows an increasing trend, rate is still lower than 50%. In 2010, a survey of 2,354 babies in 12 provinces (including Shanxi) in the central and western regions of China showed that only 28.7% of the infants received EBF within 6 months postpartum (8). A survey of 976 pregnant women in Nanchang, a city in the central region of China, from 2011 to 2013 showed that the rates of EBF were 36.0% and 34.5% at 1 and 6 months postpartum (9). In 2012, a postpartum feeding survey of 528 women in 9 counties in Zhejiang Province showed that the rate of EBF for babies at 6 months was 30.3% (10). In 2014, the EBF rate of infants in Changsha, a city in the central region of China, during hospitalization was 37.5%, and that of infants at 6 months after discharge was 40.0% (11). In 2019, a survey of 5,302 children in Zhongshan, a city in the southern region of China, showed that the EBF rate at 6 months was 42.03% (12). Lu et al. investigated the feeding status of 1,193 infants and young children in rural areas of Gansu Province from November 2018 to January 2019, and showed that the EBF rate within 6 months was 39.02% (13). Hence, breastfeeding may reduce the occurrence and mortality of pediatric infectious diseases in western China by promoting good infant and child feeding practices, particularly preventing inappropriate use of breastmilk substitutes. In addition, infant feeding habits may be related to the long-term incidence of non-communicable diseases (3). Most provinces in China has extend maternity leave by 60 days in addition to the 98 days stipulated by the state. Among them, the maternity leave in Shaanxi was extended to 158 days, and for those undergoing pre-pregnancy check-ups, it was extended to 168 days. These changes may support EBF in women from this area (14).

Therefore, this study was designed to investigate the current breastfeeding situation of full-term infants within 6 months in Xi'an and analyze its influencing factors to provide a basis for establishing breastfeeding measures and improving infant feeding in clinical practice.

## Subjects and Methods

### Study Subjects

A stratified sampling method was used to collect pregnant women and newborns after delivery during the third trimester of the study period. According to previous literature, we assumed that the EBF rate is 30%, and the formula for estimating the overall rate is as follows: $n=\frac{\mu_{1-\alpha }^{2}\pi \left(1-\pi \right)}{\delta^{2}}$.

The total number of subjects required for investigation (considering 20% loss-to-follow-up rate) was 1,609. According to the number of delivery cases of the hospitals in 2018, one provincial-level maternal and child health hospital, three municipal hospitals, and one county-level maternal and child health hospital were selected. The sample size of each hospital was calculated based on the proportion of the number of delivery cases of this hospital in all selected ones.

The full-term infants born from January 1, 2019, to February 28, 2019, and their mothers were selected as subjects at five hospitals in Xi'an, including one provincial hospital, three municipal hospitals, and one county hospital. All 5 hospitals are baby-friendly hospitals. The outpatient health education includes training sessions for pregnant women, such as the structure of breasts in different periods, breastfeeding, the process of natural childbirth, etc. After being admitted to the hospital, pregnant women were instructed with breastfeeding knowledge by the nurses. The Early Essential Newborn Care is implemented immediately after the baby is born in the delivery room. In 2 hospitals (1 at the municipal level and 1 at the county level), babies were placed on the mothers' abdomen after born and the skin contact time was no <90 min after the baby was born. In 3 hospitals (1 at the provincial level, 2 at the municipal level), the skin contact time was no <30 min. The nurses then wiped and weighed the newborns. After returning to the ward, mothers were instructed with breastfeeding practice guidance by nurses in a “one-on-one” mode. In cases with cesarean section, mothers returned to the ward, and skin contact and sucking were immediately carried out. All hospitals implemented 24-h rooming with mothers and babies. Breastfeeding consultations were provided through follow-up visits and telephone calls for 1 week after discharge.

A self-designed breastfeeding questionnaire and follow-up questionnaire were used for investigation. Eighteen hundred questionnaires with complete information were collected, all of which were continuous cases.

Inclusion criteria:

① Single-fetus full-term infants with a gestational age of at least 37 weeks; ② their parents were married and voluntarily participated in the breastfeeding investigation and were willing to cooperate with the investigators.

Exclusion criteria:

① Twins, or singles with a gestational age <37 weeks; ② their parents were unwilling to participate in the investigation; ③ mothers with infectious diseases such as HBV, HCV, etc.; ④ pregnant women participating in other studies.

### Methods

This study was an observational study, in which self-designed breastfeeding questionnaire and follow-up questionnaire were used. This study was approved by the local ethic committee and all subjects provided written informed consent before enrollment. The breastfeeding questionnaire included the demographic characteristics of pregnant women and their husbands (i.e., age, occupation, educational level, place of residence, monthly family income, etc.), general conditions of pregnant women during pregnancy (i.e., height, weight, recommendations for breastfeeding by husband and family members, planned feeding methods, complications during pregnancy, etc.), postpartum lactation-related surveys (i.e., method of delivery, current status of nipples, early initiation of breastfeeding, II lactation initiation, difficulties in feeding, etc.), and general information of babies. Before the study, a one-month pre-survey was performed in the provincial hospital from November to December 2018, and four rounds of revision of the questionnaire were completed by the chief nurses and investigators of the ward who participated in this study. The final version of the revised questionnaire was then used. The questionnaire was divided into two parts. The first part was an investigation of general information using the Breastfeeding Questionnaire, which included demographic characteristics (residence, education, family income, occupation, etc.), pregnancy (sleep during pregnancy, BMI, complications, etc.), and breastfeeding-related investigations. The second part was the follow-up investigation of newborns from birth to 6 months.

**Figure 1:**
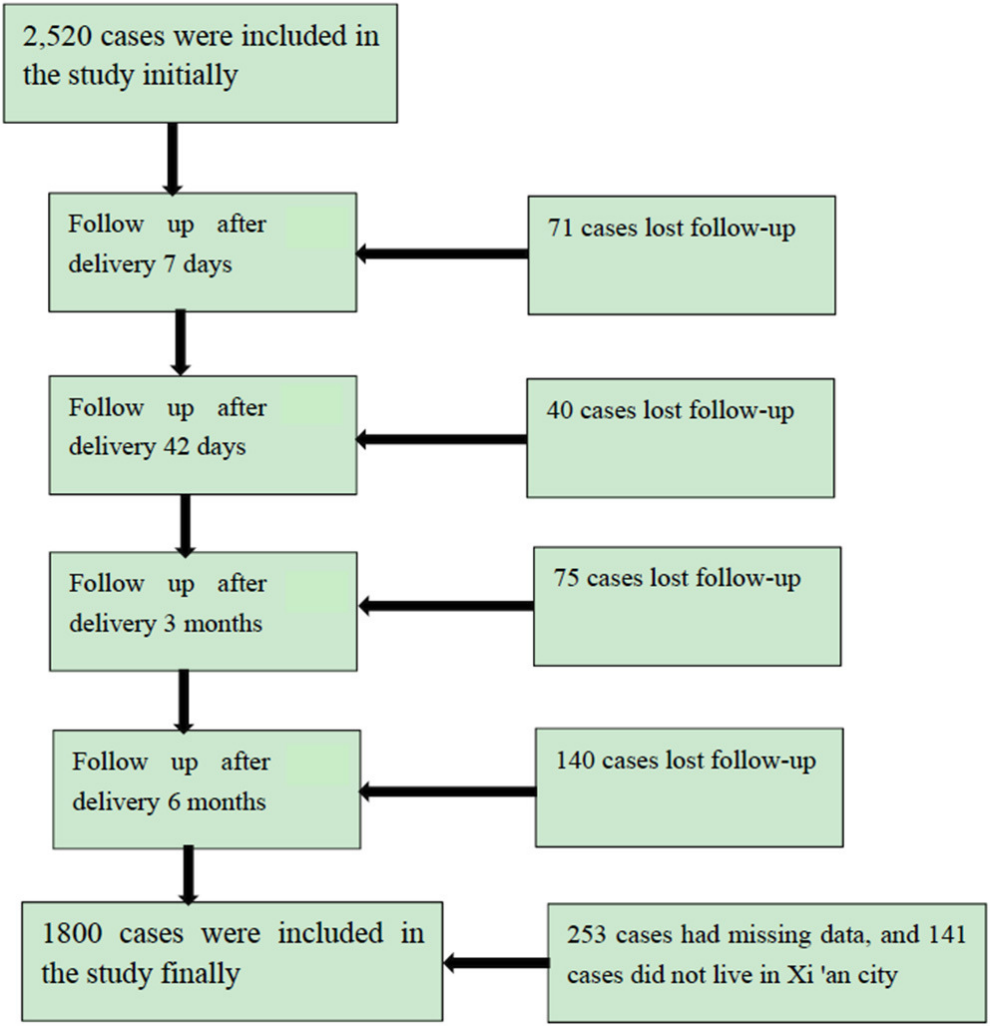
Follow-up flow chart.

A total of 2,520 full-term infants were included in this study and 1,800 cases were followed for 6 months (Figure 1). The questionnaire recovery rate was 71.43%. The baseline characteristics of 720 cases with missing data and 1,800 cases with complete follow-up data were not statistically significant (Supplementary Table 1). Therefore, the missing data did not bias the results.

### Quality Control

The pilot investigation was conducted in this study, and the questionnaire was completed at the Northwest Women's and Children's Hospital in December 2018. All team investigators received unified training, including preparations before the investigation, investigation precautions, review of questionnaires, handling of special situations, investigation methods, etc. In the on-site investigation process, the investigator maintained an objective attitude to avoid leading questions and inquired item by item according to all originally designed questionnaires, and timely checked the questionnaire. The missing and wrong items were corrected immediately once found. A double data entry method was adopted to enter the database.

### Definition of Indicators

All definitions of the terms are listed in Supplementary Table 2.

The feeding patterns of infants aged 1–6 months were divided into EBF group (EBF or occasional water intake), partial breastfeeding group (mixed feeding of breastfeeding and artificial feeding) and artificial feeding group (not Including breast milk during feeding).

According to adult BMI classification criteria for Chinese, <18.5 kg/m^2^ indicates underweight, 18.5–23.9 kg/m^2^ indicates normal weight, 24.0–27.9 kg/m^2^ indicates overweight, and ≥ 28 kg/m^2^ indicates obesity (15).

According to the Guidelines of the Institute of Medicine 2009, the recommended weight gain for underweight, normal weight, overweight and obesity are 12.5–18.0, 11.5–16.0, 7.0–11.5, and 5.0–9.0 kg, respectively. Those who fall below, meet, and exceed the above-mentioned recommended standards are, respectively, insufficient gestational weight gain (GWG), normal GWG, and excessive GWG (16).

The pain score was calculated using the Visual Analog Scale (VAS), which assessed the pain based on the subject's own perspective. The total score of the scale is 0–10 points: 1–3, mild pain; 4–6, moderate pain; 7–10, severe pain.

EBF means that the baby should not have other food or drinks except breastmilk and is allowed to take vitamins, minerals, and medicine, except for water intake.

The EBF rate = the number of infants in the same period, still receiving EBF 24 h before the investigation / number of infants in the same period ×100%.

The feeding patterns of infants aged from 1 to 6 months in this study were divided into the EBF group (EBF or occasional water intake), the partial breastfeeding group (a combination of breastfeeding and artificial feeding), and the artificial feeding group (artificial feeding only with no breastmilk included).

Pregnancy complications included gestational diabetes, pregnancy hypertension, hypothyroidism, hyperthyroidism, placenta previa, and intrahepatic cholestasis.

During pregnancy, attending a breastfeeding course refers to watching a breastfeeding promotion video, a breastfeeding course video, or witnessing a friend breastfeeding an infant.

Nipple retraction: in retraction Grade I, part of the nipple is inverted, with an existing nipple neck that can be easily squeezed out. The size of the nipple after being squeezed out is average. In retraction Grade II, the nipple is completely inverted in the areola, but the nipple can be squeezed out by hand. The nipple is smaller than average, most without a nipple neck. In retraction Grade III, the nipple is completely buried in the areola, making the inverted nipple unable to be squeezed out (17).

Pre-pregnancy BMI was calculated as follows: pre-pregnancy BMI = pre-pregnancy weight (kg)/[height (m)^2^].

According to the classification criteria of being overweight and obese in Chinese adults, BMI <18.5 kg/m^2^ is considered underweight, 18.6–23.9 kg/m^2^ is considered average, 24.0–27.9 kg/m^2^ is considered overweight, and ≥28 kg/m^2^ is considered obese (15).

Weight gain during pregnancy was calculated as follows: weight gain during pregnancy = prenatal weight (kg) – pre-pregnancy weight (kg).

According to the 2009 Institute of Medicine guidelines, the recommended weight gain standards for underweight, average weight, overweight, and obese women were 12.5–18.0 kg, 11.5–16.0 kg, 7.0–11.5 kg, and 5.0–9.0 kg, respectively. The weight lower than, in line with, and higher than the above-recommended standards was considered insufficient GWG, normal GWG, and excessive GWG, respectively (16).

The postpartum breastfeeding pain score in 48 h: mild pain (1–3), moderate pain (4–6), and severe pain (7–10).

### Statistical Analysis

EpiData 3.0 was used to establish the database by double data entry. The classified data were expressed as the rate, and the continuous data were expressed as mean ± standard deviation. Univariate analysis was performed using the survival analysis K–M method, and multivariate analysis was performed using the Cox survival risk model. Before applying the Cox proportional hazard model, the time-dependent model was used to verify the proportional risk of the above factors, which all met the equal proportional risk hypothesis. Therefore, the Cox regression was included. All statistical analyses were performed using SPSS 22.0 software. For bilateral inspection, the inspection level was set as α = 0.05.

## Results

### Investigations of General Demographic Data

This study was a descriptive study in a non-experimental manner. The distribution of puerpera in this study was 925 cases (51.38%) at provincial hospitals, 645 patients (35.83%) at municipal hospitals, 230 cases (12.78%) at county health hospitals. The mean pre-pregnancy BMI was 21.50 ± 3.08 kg/m^2^, and in the third trimester was 26.71 ± 3.43 kg/m^2^, with an average weight increase of 13.63 ± 5.40 kg. The mothers and their husbands' occupations' were mostly company employees, accounting for 34.05% and 40.00%, respectively. There were 758 people with maternity leave ≥150 days, accounting for 42.11%. Complications occurred in 678 people (37.67%). The four primary complications were 275 (15.27%) with gestational diabetes, 142 (7.89%) with hypothyroidism, 85 (4.72%) with placenta previa, and 49 (2.72%) with hypertension during pregnancy. See Table 1 for the general demographic data of parturients and newborns.

**Table 1:** Demographic and clinical data of months and newborns.

**Item**	**Number of cases (%) unless indicated elsewhere**
Age (Years)	
21–25	188 (10.44%)
26–30	889 (49.38%)
30–35	577 (32.05%)
>35	146 (8.11%)
History of maternity	
Primipara	630 (35.00%)
Multipara	1,170 (65.00%)
Monthly household income (RMB/month)	
≤ 5,000	402 (22.33%)
5,001–10,000	811 (45.06%)
≥10,000	587 (32.61%)
Educational Level	
College and below	932 (51.77%)
Bachelor degree	688 (38.22%)
Master degree or above	182 (10.11%)
Residence location	
Urban	1,087 (60.39%)
Suburban	713 (39.61%)
Medical payment type	
Medical insurance	1,543 (85.72%)
Self-funded	257 (14.28%)
Complications	
No	1,122 (62.33%)
Yes	678 (37.66%)
Breastfeeding courses involved during pregnancy	
No	1,155 (64.17%)
Yes	645 (35.83%)
Baby's sex	
Male	927 (51.50%)
Female	873 (48.50%)
Gestational age in weeks	39.31 ± 1.84W;
Body weight (Kg)	3.62 ± 1.91kg
Early initiation of breastfeeding	826 (45.89%)
Admission to neonatology department after birth	236 (13.11%)

*The RMB is trading at 6.435 to the US dollar.

### Investigation on the Current Situation of Postpartum Lactation

An investigation was made on the present condition of postpartum mothers' breasts, initiation of lactation, lactation pain, and cracked nipples. The results showed that 21.72% of the parturients had initiation of postpartum lactation stage II. A total of 13.22% of parturients suffered from nipple depression, and Grade I retraction was dominant, accounting for 67.64%. A total of 91.83% of parturients had pain during lactation. There were 281 cases (15.61%) of cracked nipples due to neonatal sucking. Within 24 to 48 h postpartum, 1,607 (89.27%) mothers faced problems related to postpartum breastfeeding. Among them, 694 (43,19%) neonates could not be fed effectively; 665 (41.38%) mothers had wound pain and had inconvenience to turn over; 598 (37.21%) neonates were difficult to wake up; 439 (27.32%) mothers had incorrect feeding posture; 181 (11.26%) mothers experienced other problems (see Table 2).

**Table 2:** The postpartum lactation of mothers (*n* %).

**Items**	**Number of cases (%)**
Start of lactation stage II	391 (21.72%)
Nipple retraction	238 (13.22%)
Retraction Grade 1	161 (67.64%)
Retraction Grade 2	48 (20.17%)
Retraction Grade 3	5 (2.10%)
Postpartum lactation pain	1,653 (91.83%)
Mild	1,325 (80.16%)
Moderate	321 (19.42%)
Severe	7 (0.42%)
Cracked nipple (evaluated at 24 – 48 h postpartum)	281 (15.61%)
Issues related to postpartum breastfeeding	1,607 (89.27%)
Baby cannot be fed effectively	694 (43.19%)
Wound pain, inconvenience to turn over	665 (41.38%)
Difficult to wake up the baby	598 (37.21%)
Incorrect feeding posture	439 (27.32%)
Others	181 (11.26%)

### Postpartum Follow-Up Survey

We further investigated the reasons why infants were fed with formula powder during follow-up. At 10 days, 42 days, 3 months, and 6 months postpartum, the follow-up data were recorded. As shown in Table 3, the rate of insufficient breast milk gradually decreased, while the rate of work gradually increased. Insufficient breast milk and work (including out-of-office work and non-office work) remained the major reasons for adding formula powder to infant feeding.

**Table 3:** The reasons of formula powder feeding during follow-up [*n* (%)].

**Postpartum time points**	**Insufficnt breast milk**	**Work**	**Medication**	**Cracked nipples**	**Infant hospitalization**	**Others**
10 days	528 (29.33%)	48 (2.67%)	22 (1.22%)	26 (1.44%)	22 (1.22%)	49 (2.72%)
42 days	501 (27.83%)	96 (5.33%)	21 (1.17%)	73 (4.06%)	21 (1.17%)	102 (5.67%)
3 months	439 (24.39%)	249 (13.83%)	16 (0.89%)	10 (0.56%)	32 (1.78%)	175 (9.72%)
6 months	322 (17.89%)	492 (27.33%)	18 (1.00%)	10 (0.56%)	25 (1.39%)	235 (13.01%)
Total	1,790 (50.68%)	885 (25.06%)	77 (2.18%)	119 (3.37%)	100 (2.83%)	561 (15.88%)

### Survival Analysis of EBF Rates

#### Comparison of Breastfeeding Rates in Different Periods

**Figure 2:**
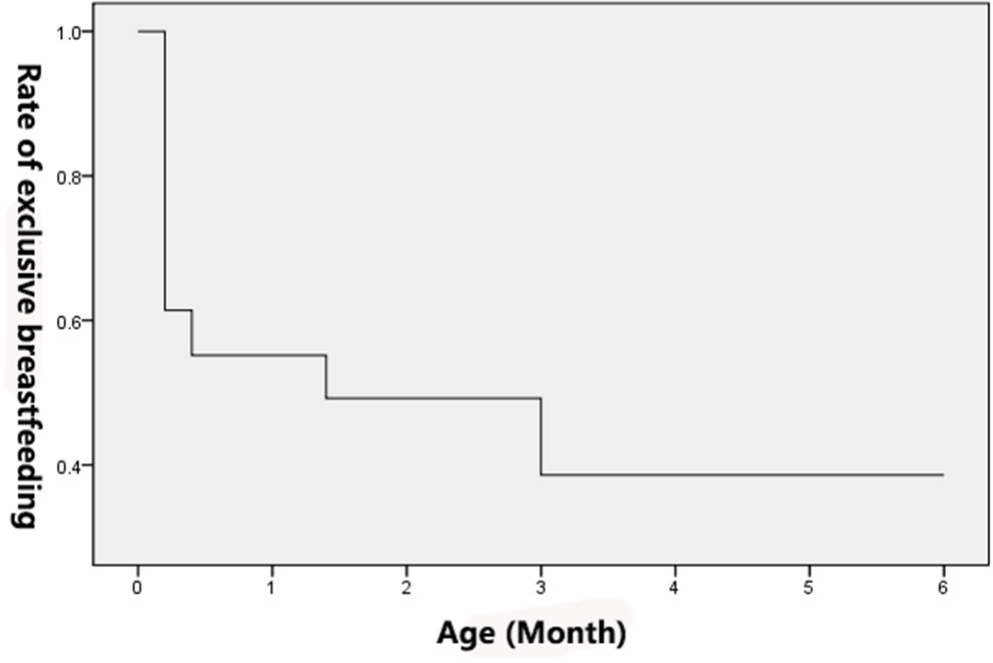
Curve of exclusive breastfeeding rate.

Postpartum follow-up showed that the rate of EBF decreased from 61.39% to 38.78% over time, showing a decreasing trend, as shown in Table 4. With the duration of EBF as the X-axis and the cessation of EBF as the outcome (Y-axis), survival analysis showed that the rate of EBF decreased more significantly after 3 months (Figure 2).

**Table 4:** Breastfeeding investigation on full-term infants within 6 months.

**Time**	**Exclusive breastfeeding rate (n, %)**	**Partially exclusive breastfeeding rate (n, %)**	**Breastmilk substitutes only (n, %)**
10 days after delivery	1,105 (61.39%)	667 (37.05%)	28 (1.56%)
42 days after delivery	986 (54.78%)	768 (42.67%)	46 (2.56%)
3 months after delivery	879 (48.83%)	845 (46.94%)	76 (4.22%)
6 months afterdelivery	698 (38.78%)	989 (54.94%)	113 (6.28%)

#### Univariate Survival Analysis of Breastfeeding (K–M Method)

Whether the infant was exclusively breastfed within 6 months was taken as the outcome variable Y (EBF = 0, non-EBF = 1). The univariate survival analysis (K–M method) was performed using 16 factors, including age, pre-pregnancy BMI, pregnancy weight gain, parturients' education, husbands' education, family monthly income, residence, whether primiparae, the mode of delivery, complications during pregnancy, feeding suggestions for husband, feeding suggestions for family members, participation in breastfeeding courses during pregnancy, the frequency of daily breastfeeding 48 h after delivery, the planned breastfeeding mode 6 months after delivery, and the scheduled breastfeeding time (Table 5). The results showed statistical differences in 12 factors among the groups, including pregnancy weight gain, parturients' education, husbands' education, family monthly income, residence, mode of delivery, feeding suggestions for husband and family members, participation in breastfeeding courses during pregnancy, the number of daily breastfeeding 48 h after delivery, the planned breastfeeding mode 6 months after delivery, and the scheduled breastfeeding time.

**Table 5:** K-M analysis of exclusive breastfeeding in full-term infants within six months.

**Items**	**Exclusive breastfeeding (n, %)**	**Non-exclusive breastfeeding (n, %)**	***χ^2^***	***P***
Weight gain during pregnancy			19.76	0.000
Standards-compliant	293 (41.79%)	408 (58.21%)		
Below standard	227 (42.19%)	311 (57.81%)		
Above standard	175 (31.19%)	386 (68.81%)		
Monthly household income (RMB/month)				
≤ 5,000	126 (32.06%)	267 (67.94%)	10.51	0.005
5,001–10,000	316 (38.63%)	502 (61.37%)		
≥10,000	253 (42.95%)	336 (57.05%)		
Resident location				
Urban	488 (44.89%)	599 (55.11%)	38.84	0.000
Suburban	207 (29.03%)	506 (70.97%)		
Mothers' education level				
College or below	310 (33.26%)	622 (66.74%)	28.91	0.000
Bachelor degree	295 (43.00%)	391 (57.00%)		
Master degree or above	90 (49.45%)	92 (50.55%)		
Husbands' education level				
College or below	296 (33.98%)	575 (66.02%)	15.57	0.000
Bachelor degree	303 (42.73%)	406 (57.27%)		
Master degree or above	96 (43.63%)	124 (56.37%)		
Mode of delivery				
Vaginal delivery	362 (40.44%)	533 (59.56%)	4.51	0.034
Cesarean delivery	333 (36.79%)	572 (63.21%)		
Husbands' advice on feeding				
Exclusive breastfeeding	609 (39.96%)	915 (60.04%)	8.29	0.004
Non-exclusive breastfeeding	86 (31.16%)	190 (68.84%)		
Family' advice on feeding				
Exclusive breastfeeding	606 (39.92%)	912 (60.08%)	7.53	0.006
Non-exclusive breastfeeding	89 (31.56%)	193 (68.44%)		
Whether to participate in breastfeeding courses				
N	375 (32.47%)	780 (67.53%)	55.42	0.000
Y	320 (49.61%)	325 (50.39%)		
Times of daily breastfeeding 48 h postpartum				
6–8 times	170 (30.69%)	384 (69.31%)	50.07	0.000
<6 times	327 (38.11%)	531 (61.89%)		
>8 times	198 (51.03%)	190 (48.97%)		
Planned breastfeeding time			21.64	0.000
<6 months	11 (23.40%)	36 (76.60%)		
6–12 months	300 (35.67%)	541 (64.33%)		
>12 months	352 (43.51%)	457 (41.49%)		
Not confirmed	32 (31.07%)	71 (68.93%)		
Breastfeeding mode six months after the delivery				
Exclusive breastfeeding	438 (41.48%)	618 (58.52%)	11.10	0.001
Non-exclusive breastfeeding	257 (34.54%)	487 (65.45%)		

#### Multivariate Survival Analysis of Breastfeeding

Whether the infant was exclusively breastfed within 6 months was taken as the outcome variable Y (EBF = 0, and non-EBF = 1). The above 16 factors were subjected to multivariate Cox regression, with the assigned values shown in Table 6. Stepwise regression analysis showed that factors affecting EBF within 6 months were weight gain during pregnancy higher than recommended, residence in the city, participation in breastfeeding courses during pregnancy, and the frequency of daily breastfeeding 48 h after delivery of more than eight times. From the sign of regression coefficient and relative risk grade, weight gain during pregnancy higher than the recommended standard and residence in suburban counties were risk factors, while parturients with a postgraduate education or above, attend breastfeeding classes during pregnancy, breastfeeding more than eight times per day after delivery were protective factors. The risk of stopping EBF within 6 months after delivery for the parturients: (1) whose weight gains during pregnancy were higher than the recommended standard was 1.186 times higher than those whose weight gains met the recommended criteria; (2) living in suburban counties was 1.219 times higher than those living in cities.; (3) those who participated in breastfeeding courses during pregnancy were 0.73 times higher than those who did not. Parturients (with daily breastfeeding more than eight times in 48 h after delivery) had a 0.66-fold risk of ending EBF 6 months after delivery, compared with those who breastfed less than six times daily (Table 6).

**Table 6:** Cox proportional hazard model.

**Independent variable**	**β**	**S.E**	**Wald**	***P***	***HR***	**95% CI**	
						**Lower limit**	**Upper limit**
Weight gain during pregnancy conforming to the standard	1	1	1	1	1		
< standard	0.015	0.076	0.037	0.847	1.015	0.874	1.178
> standard	0.171	0.073	5.436	0.020	1.186	1.028	1.369
Resident location	0.198	0.067	8.686	0.003	1.219	1.069	1.391
Breastfeeding courses during pregnancy	−0.308	0.070	19.318	0.000	0.735	0.641	0.843
Times of daily breastfeeding 48 h after delivery <6 times	1	1	1	1	1		
6–8 times	−0.16	0.068	3.451	0.063	0.881	0.771	1.007
More than 8 time	−0.47	0.091	19.99	0.000	0.665	0.557	0.796

## Discussion

A cohort study in northwest China from 2007 to 2010 showed that the EBF rate of newborns after 14 days was 24%, and after six months, only 3% (18). A summary analysis of breastfeeding rates in China from 2007 to 2018 reported that in Xi'an, the EBF rates of 3,580 infants at 1, 3, and 6 months were 76.51%, 47.09%, and 16.31%, respectively (19). China's breastfeeding data (UNICEF data, 2012–2014) showed that the EBF rate within 6 months was 28%, which has not increased in the past 3 years (20–22). In recent years, with the publicity and promotion of breastfeeding knowledge in baby-friendly hospitals and the extension of maternity leave, more parturients have joined in the learning and practice of breastfeeding knowledge, and the rate of EBF has continuously increased. This study showed that the EBF rate of full-term infants within 3 and 6 months was 48.83% and 38.78%, which was higher than the reported data and might be related to the selection of full-term infants in this study. Zong et al. (23) showed that the EBF rate within 6 months in nine cities in China was 48.6%, which was higher than this study, indicating that breastfeeding education is still required to be strengthened in those regions for further improvement.

The systematic evaluation by Cohen et al. showed that the influencing factors for the initiation and continuation of breastfeeding mainly included smoking, mode of delivery, multiple births, parturients' education, and breastfeeding education (24). This study showed that breastfeeding courses during pregnancy were a protective factor for EBF 6 months after delivery, which was 0.73 times higher than the non-EBF rate of parturients who did not receive breastfeeding courses. This study showed that parturients after delivery suffered various difficulties in breastfeeding. For this reason, breastfeeding instruction should be provided to parturients throughout the postnatal period until they are comfortable and keep breastfeeding. For example, after delivery, attention should be paid to the parturients' different degrees of lactation pain and take corresponding measures to reduce pain and the incidences of postpartum cracked nipples, prolonging the lactation time. Concern should be given to the initiation time of parturients lactation in the early postpartum period, starting lactation as early as possible through the effective sucking of the infant to ensure the lactation amount was conducive to promoting breastfeeding.

The results of this study showed that when the parturients' weight gains during pregnancy with different BMI ranges before pregnancy were higher than the recommended standards, the risk of non-EBF within 6 months after delivery was 1.186 times higher than those with weight gains meeting the recommended standards. Huang (25) found that excessive weight gain during pregnancy increased the risk of premature termination of breastfeeding, and the risk of delayed lactation initiation for parturients with excessive weight gain increased by 10%. Huang et al. (26) conducted a systematic evaluation of the literature before February 2019. The results showed that pre-pregnancy obesity was a risk factor for breastfeeding initiation, EBF, and the duration of any breastfeeding. Excessive weight gain or obesity during pregnancy were risk factors for the duration of EBF. Therefore, women of childbearing age who were obese before pregnancy or had too much or not enough weight gain during pregnancy were significantly less likely to initiate breastfeeding and continue to breastfeed for the recommended duration. In a study of low-income women in the United States, Li et al. (27) found that the mother's pre-pregnancy BMI, being overweight or obese, and pregnancy weight gain were independent factors affecting the length of breastfeeding; there was no correlation between them. Hilson et al. (28) found no correlation between pre-pregnant BMI and GWG on EBF and breastfeeding in a study of 2,783 breastfeeding women in the United States. In this study, there was still no correlation between pre-pregnancy BMI and GWG. Weight management is an essential part of health care during pregnancy. Therefore, proper weight control of women of childbearing age and keeping their weight gain within the normal range during pregnancy can promote EBF and prolong the breastfeeding time.

Since the “two-child policy,” the state has advocated breastfeeding to reduce infant mortality and improve infant health conditions. The latest study shows that the infant mortality rate (<1 years-old/thousand) in rural areas is more than twice that in urban areas (29). Other studies have also shown that the growth of infants and children in rural areas lags behind that in urban areas, and poor infant feeding practices, including inadequate breastfeeding, are prevalent in these areas (30–33). In this study, the EBF rate was 44.89% within 6 months for urban areas and 29.03% for suburban counties (*P* = 0.00), with a statistically significant difference. Due to the imbalance in economic and educational development between cities and suburban counties, the difference in the acceptance and execution of the concept of feeding among the population, and the publicity of various milk powder advertisements, the belief in EBF is easily shaken, thus reducing the rate of EBF in suburban counties. Therefore, it is necessary to take appropriate intervention measures to improve the knowledge and practices of parturients and family members on breastfeeding in suburban areas to increase the rate of EBF and prolong breastfeeding time.

This study mainly investigated the EBF rate of full-term infants within 6 months in the Xi'an area and analyzed its influencing factors to provide the basis for the clinical designation of targeted measures. However, during the research, due to the adjustment of obstetrics work mode in some hospitals, the final collected data was less than the planned cases. After the unqualified samples were excluded, the qualified samples were fewer. A total of 326 parturients were lost within the 6-month follow-up period with a rate of 12.9%. Most parturients were lost to follow-up in the sixth month, which might be related to the decrease of parturients' cooperation with a prolonged study. The above two points may limit the outcome of this study.

## Conclusion

Our work fills the gap in the rate of exclusive breastfeeding for full-term infants in Xi'an, China. This study showed that the EBF rates in Xi'an at 10 days, 42 days, 3 months, and 6 months after birth were 61.39%, 54.78%, 48.83%, and 38.78%, respectively. There is a downward trend over time. Although the EBF rate within 6 months has increased compared with previous reported data, there is still a gap with the WHO's target of 50% within 6 months. Our study also found that weight gain during pregnancy higher than the recommended standard, and living in a suburban county was a risk factor for mothers to terminate exclusive breastfeeding within 6 months after delivery, while taking breastfeeding courses during pregnancy, and the number of breastfeeds per day within 48 hours after delivery was >8 were the factors contributing to continuous EBF for 6 months postpartum. With the changes in people's eating habits and dietary structure, excessive intake of fats and oils during pregnancy may lead to excessive weight gain and excessive weight gain, and may cause early termination of EBF after delivery. Wang et al. investigated postpartum lactation in 240 parturients and found that high BMI and high GWG before pregnancy are risk factors for delayed lactation initiation. Primiparas were more prone to delayed onset of lactogenesis (34). It has also been shown that the breastfeeding rate of women with high pre-pregnancy BMI and excessive weight gain during pregnancy decreased the breastfeeding rate of women (35). In addition, excessive weight gain during pregnancy also increases the risk of pregnant women developing gestational diabetes (36). Therefore, weight control during pregnancy is a key. After delivery, early breastfeeding (early contact, early sucking, early milking) should be recommended to promote the initiation of the second stage of lactation. More attention should be paid on mothers facing breastfeeding problems. In addition, by using community medical resources, publicity of breastfeeding for pregnant women in suburbs and counties should be recommended. Future investigations are needed to explore the effects of the spouses and other family members in breastfeeding to establish a family-style breastfeeding support system should be established.

## Data Availability Statement

The original contributions presented in the study are included in the article/Supplementary Material, further inquiries can be directed to the corresponding author/s.

## Ethics Statement

The studies involving human participants were reviewed and approved by Ethics Committee of Northwest Women's and Children's Hospital (20-078). Written informed consent to participate in this study was provided by the participants' legal guardian/next of kin.

## Author Contributions

LM, W-LG, and Z-WY: conception and design of the research. X-YL, X-QZ, J-JZ, and JY: acquisition of data. Z-WY: analysis and interpretation of the data and writing of the manuscript. Z-WY and P-FQ: statistical analysis. LM: obtaining financing and critical revision of the manuscript for intellectual content. All authors read and approved the final draft.

## Funding

This study was funded by Shaanxi Province Key Research and Development Program General Project–Social Development (2018SF-224). The funding body had no role in the design of the study and collection, analysis, and interpretation of data and in writing the manuscript.

## Conflict of Interest

The authors declare that the research was conducted in the absence of any commercial or financial relationships that could be construed as a potential conflict of interest.

## Publisher's Note

All claims expressed in this article are solely those of the authors and do not necessarily represent those of their affiliated organizations, or those of the publisher, the editors and the reviewers. Any product that may be evaluated in this article, or claim that may be made by its manufacturer, is not guaranteed or endorsed by the publisher.
