# Supplementary material for: The Influencing Factors of Breastfeeding for Full-Term Singleton Within Six Months of Birth in Xi'an Before the Covid-19 Pandemic
Source: Front Pediatr. 2022 Mar 10;9:801436. doi: 10.3389/fped.2021.801436 (PMC8961653; doi:10.3389/fped.2021.801436)
Supplement: Supplementary file 1 [file Table_1.docx]

**Supplementary Table 1. Cases with complete follow-up data or missing data**

| **Items** | **Cases with complete follow-up data**  **n (%)** | **Cases with**  **missing data**  **n (%)** | ***χ^2^*** | ***P*** |
| --- | --- | --- | --- | --- |
| **Age (years)** |  |  |  |  |
| 21-25 | 188 (10.44%) | 77 (10.69%) | 4.78 | 0.188 |
| 26-30 | 889 (49.38%) | 381 (52.92%) |  |  |
| 30-35 | 577 (32.05%) | 199 (27.64%) |  |  |
| >35 | 146 (8.11%) | 63 (8.75%) |  |  |
| **History of maternity** |  |  |  |  |
| Primipara | 630 (35.00%) | 271 (37.64%) | 1.559 | 0.212 |
| Multipara | 1170 (65.00%) | 449 (62.36%) |  |  |
| **Monthly household income (RMB/month)** |  |  |  |  |
| ≤5000 | 402 (22.33%) | 162 (22.50%) | 0.298 | 0.862 |
| 5001-10000 | 811 (45.06%) | 330 (45.83%) |  |  |
| ≥10000 | 587 (32.61%) | 228 (31.67%) |  |  |
| **Education level** |  |  |  |  |
| Collage and below | 932 (51.77%) | 344 (47.78%) | 5.525 | 0.063 |
| Bachelor degree | 688 (38.22%) | 283 (39.31%) |  |  |
| Master degree and above | 182 (10.11%) | 93 (12.91%) |  |  |
| **Residence location** |  |  |  |  |
| Urban | 1087 (60.39%) | 455 (63.19%) | 1.705 | 0.192 |
| Suburban | 713 (39.61%) | 265 (36.81%) |  |  |
| **Medical payment type** |  |  |  |  |
| Medical insurance | 1543 (85.72%) | 601 (83.47%) | 2.051 | 0.152 |
| Self-funded | 257 (14.28%) | 119 (16.53%) |  |  |
| **Complications** |  |  |  |  |
| No | 1122 (62.33%) | 443 (61.53%) | 0.142 | 0.706 |
| Yes | 678 (37.66%) | 277 (38.47%) |  |  |
