# Supplementary material for: The Influencing Factors of Breastfeeding for Full-Term Singleton Within Six Months of Birth in Xi'an Before the Covid-19 Pandemic
Source: Front Pediatr. 2022 Mar 10;9:801436. doi: 10.3389/fped.2021.801436 (PMC8961653; doi:10.3389/fped.2021.801436)
Supplement: Supplementary file 2 [file Table_2.docx]

**Supplementary Figure 2. Definition of terms**

| **Item** | **Definition** |
| --- | --- |
| Exclusive breastfeeding | baby should not have any food or drink other than breast milk. Vitamins, minerals and drugs are allowed, but water is not included ^[6]^ |
| Exclusive breastfeeding rate | The number of infants in the same period who were still receiving exclusive breastfeeding 24 hours before the survey/the number of infants in the same period × 100% |
| Complications during pregnancy | Including gestational diabetes, pregnancy-induced hypertension, hypothyroidism, hyperthyroidism, placenta previa, cholestasis syndrome |
| Nipple condition assessment | Large nipples: 16 – 23 mm  Extra-large nipples: >23 mm |
| Inverted nipples | Degree I: part of the nipple is inverted, and the nipple neck is present, which can be easily squeezed out. The size of the nipple after being squeezed out is similar to that of ordinary people;  Degree II: The nipple is completely sunken in the areola, but the nipple can be squeezed out by hand. The nipple is smaller than normal, and most of it has no nipple neck;  Degree III: The nipple is completely buried under the areola, and the inverted nipple cannot be squeezed out [7] |
| Participate in breastfeeding courses during pregnancy | have watched breastfeeding videos, breastfeeding course videos, or have seen a friend feed a baby. |
| BMI before pregnancy | Weight before pregnancy (kg)/height (m^2^) |
| Weight gain during pregnancy | Prenatal weight (kg) – pre-pregnancy weight (kg) |
